# Effects of valsartan combined with α-lipoic acid on renal function in patients with diabetic nephropathy: a systematic review and meta-analysis

**DOI:** 10.1186/s12902-021-00844-0

**Published:** 2021-08-31

**Authors:** Fangfang Sun, Deqi Jiang, Juanjuan Cai

**Affiliations:** 1 Department of pharmacy, Hangzhou Third People’s Hospital, Hangzhou, 310009 China; 2 Department of Biology and Pharmacy, Yulin Normal University, Yulin, 537000 China; 3 Department of Pathology, Zhejiang Province People’s Hospital, Hangzhou, 310014 China

**Keywords:** Diabetic nephropathy, Valsartan, α-Lipoic acid, Renal function, meta-analysis

## Abstract

**Background:**

Diabetic nephropathy (DN) is one of the most serious microvascular complications of diabetes, valsartan and α-lipoic acid alone or in combination has been used for the treatment of patients with DN. However, some results in these clinical reports were still controversial. The purpose of this study was to evaluate the efficacy of valsartan combined with α-lipoic acid on renal function in patients with DN.

**Methods:**

We searched the electronic databases including PubMed, Sciencedirect, EMBASE, Cochrane library, Chinese national knowledge infrastructure (CNKI) and Wanfang databases, and the publication deadline was limited to January 2020. Randomized controlled trials (RCTs) evaluating the effects of valsartan combined with α-lipoic acid in DN patients were included. Pooled estimates were conducted using a fixed or random effect model. The outcomes included urinary albumin excretion rate (UAER), and the level of urinary albumin, β_2_-microglobulin (β_2_-MG), hypersensitive C-reactive protein (hs-CRP) and oxidative stress.

**Results:**

11 studies with 1294 participants were included in this study. The pooled analysis indicated that α-lipoic acid combined with valsartan could remarkably reduce UAER (*P* < 0.00001, SMD = -1.95, 95%CI = -2.55 to − 1.20; *P* = 0.03, SMD = -0.85, 95%CI = -1.59 to − 0.1) and the level of urinary albumin (*P* = 0.001, SMD = -1.48, 95%CI = − 2.38 to − 0.58; *P* = 0.01, SMD = -1.67, 95%CI = -3.00 to − 0.33), β_2_-MG (*P* < 0.001,SMD = − 2.59, 95%CI = -3.78 to − 1.40; *P* = 0.03, SMD = -0.48, 95%CI = -0.93 to − 0.04) when compared with valsartan or lipoic acid monotherapy in patients with DN. However, there was no significant difference in the level of hs-CRP among the three therapies (*P* = 0.06, SMD = -2.80, 95%CI = -5.67 to 0.07; *P* = 0.10, SMD = -0.42, 95%CI = − 0.92 to 0.08). In addition, α-lipoic acid combined with valsartan markedly increased the level of SOD (*P* = 0.03, SMD = 1.24, 95%CI = 0.32 to 1.03; *P* = 0.0002, SMD = 0.68, 95%CI = 0.32 to 1.03) and T-AOC (*P* < 0.00001, SMD = 0.89, 95%CI = 0.62 to 1.16; *P* = 0.02, SMD = 0.58, 95%CI = 0.10 to1.07), and reduced the level of MDA(*P* = 0.0002, SMD = -1.99, 95%CI = -3.02 to − 0.96; *P* = 0.0001, SMD = -0.69, 95%CI = -1.04 to − 0.34).

**Conclusions:**

α-lipoic acid combined with valsartan could significantly reduce the level of urinary albumin and oxidative stress, increase antioxidant capacity and alleviate renal function damage in patients with DN, and this will provide a reference for the selection of treatment drugs for DN.

## Background

Diabetic nephropathy accounts for 20–30% of patients with diabetes mellitus (DM), which is a frequent microvascular complication of DM and one of the important causes of chronic renal failure [1]. At present, about 15% of DN patients would develop end-stage renal disease, and eventually lead to death due to renal failure [2]. Most patients with DN have an insidious onset, no obvious clinical symptoms in the initial stage, but the disease deteriorates when persistent albuminuria emerges. The pathophysiologic mechanisms of DN are multi-factorial. Previous studies found that abnormal hemodynamics, oxidative stress response, genetic factors, activation of protein kinase C and renin angiotensin II, and pro-inflammatory reactions were all related to the occurrence and progression of DN [3]. Hyperglycemia in DM could lead to a series of metabolic, hemodynamic and biochemical alterations in renal tissues, including the increasing formation of advanced glycation end products and reactive oxygen species (ROS), extracellular matrix accumulation, growth factors/cytokines secretion, alteration in glomerular filtration rate which causes proteinuria, and all these changes might lead to DN [4]. In addition, hyperglycemia could increase the production of renin angiotensin II, resulting in the profibrogenic and inflammation of renal tissue [5]. Moreover, the progression of DN could heighten the risk of morbidity and mortality of cardiovascular diseases.

At present, a variety of treatments, including lowering blood sugar, blood pressure and blood fat, anti-inflammatory, anti-oxidation, traditional Chinese medicine treatment, combination of traditional Chinese and western medicine, were all used to mitigate renal function damages in patients with DN. However, the drugs prove effective against DN are still limited. Clinically, the commonly used therapeutic drugs included angiotensin converting enzyme inhibitors, angiotensin II receptor antagonists, HMG-CoA reductase inhibitors, vitamin D and its analogs, protein kinase C inhibitors, antioxidants, etc. These drugs used alone or in combination could reduce the level of proteinuria and improved kidney function. In addition, sodium-glucose cotransporter 2(SGLT2) inhibitors had also been used to reduce the levels of microalbuminuria and albuminuria due to its anti-proteinuric effects [6].

Valsartan is a kind of angiotensin II receptor blockers (ARBs), which has been widely used in the treatment of hypertension and showed renoprotective and cardioprotective effects [7]. Valsartan could antagonize the binding of angiotensin II to its receptor, dilate the glomerular artery blood vessels and lower glomerular pressure, reducing protein filtration and inhibiting the production of proteinuria [8]. In addition, valsartan could down-regulate the expression of endothelin and cytokines, inhibit the proliferation of glomerular cells, promote the degradation of extracellular matrix and suppress the collagen synthesis, then delay glomerular sclerosis, thus boosting renal function [9]. Results in one RCT indicated that valsartan could lower the incidence of microalbuminuria, and without increasing the incidence of renal dysfunction in patients with glucose tolerance [10]. In streptozotocin and high fat diet induced DN mice, valsartan could alleviate renal podocyte injure and inhibit the expression of profibrotic growth factors and proinflammatory cytokines, decrease the accumulation of lipids and the production of albuminuria, improve glomerulosclerosis [11]. Moreover, valsartan combined with other drugs (such as angiotensinase inhibitors, antioxidants, prostaglandin E1, etc.) could also significantly reduce proteinuria and alleviate kidney damage in diabetic patients [12].

Lipoic acid or α-lipoic acid is a powerful antioxidant that can remove a variety of oxidative stress products including hydroxyl radicals, singlet oxygen, nitric oxide radicals, hydroperoxides, hydroperoxides, and regenerate other antioxidants (such as vitamins C, vitamins and glutathione) to maintain normal antioxidant capacity [13]. Previous study indicated that lipoic acid could up-regulate the expression of Nrf-2-mediated antioxidant genes and peroxisome proliferator activated receptors-regulated genes, enhance the antioxidant defense system, and had been used for the treatment of diabetic complications [14]. In mitochondria, lipoic acid synthase could produce α-lipoic acid that participates in regulating the process of glucose oxidation and ATP generation, while reduction of lipoic acid synthase would increase oxidative stress response and accelerate the progression of DN [15]. Further study found that lipoic acid could lower the concentration of proteinuria and mitigate oxidative stress and renal damage in diabetic rats [16]. One RCT reported that lipoic acid combined with pyridoxine could reduce oxidative stress and albuminuria in patients with DN [17].

In addition, the combination treatment of lipoic acid and ARBs (such as valsartan, telmisartan, losartan) has been used to bolster the renal function in patients with DN. However, the combined effects of these agents were still controversial. Moreover, few studies assess the effects of telmisartan or losartan combined with lipoic acid in the treatment of DN, while some clinical trials about the combination treatment of lipoic acid and valsartan have been reported. Therefore, we performed this meta-analysis to evaluate the efficacy of valsartan combined with lipoic acid on renal function in patients with DN.

## Methods

### Literature search

We conducted a systematic literature search according to the PRISMA guidelines, and the electronic databases included PubMed, Sciencedirect, Embase, Cochrane library, CNKI and Wanfang databases. All the databases were searched without language restrictions, and the studies were published up to January 2020. The keywords included valsartan or angiotensin II receptor blockers or ARBs, lipoic acid or α-lipoic acid, DN or diabetes kidney disease, and randomized controlled trials.

### Study selection and exclusion criteria

The included studies met the following criteria: a) patients were diagnosed with DN, b) the study designed to be a randomized controlled clinical trial, c) the studies evaluated the effects of valsartan combined with lipoic acid on renal functions, d) with treatment duration of at least 14 days, and full-text publications were available. The exclusion criteria are as follows: patients were diagnosed with DN and other complications; non-RCTs and protocol; studies with insufficient data.

### Data extraction

All relevant data were separated using a customized data extraction table and performed by two independent reviewers. The information of extraction table included first authors’ names and publication dates, types of studies, numbers of patients enrolled, the base characteristics of patients (including age and sex), interventions, duration of medication, dosage of valsartan and lipoic acid, and Jadad score. The clinical outcomes included UAER, urinary albumin, β_2_–MG, hs-CRP, SOD, malondialdehyde (MDA) and total antioxidant capacity(T-AOC).

### Quality assessment

The Jadad scale was used to evaluate the quality of each selected study. The Jadad scores of the study ranged from 0 to 7 points, and the main contents included the methods of randomization, double blinding, allocation concealment, withdraw and dropouts. Any disagreements were resolved by discussion.

### Statistical analysis

The data analysis was conducted using RevMan software 5.3, and random or fixed effect models were used to analyze the outcomes. The heterogeneity between studies was evaluated using the *I*^*2*^ test. Significant heterogeneity was identified and random effect model was used when *I*^*2*^ ≥ 50% or *P*-value ≤0.05. Otherwise the heterogeneity was low and fixed effect model was applied. For continuous outcomes, data were represented as standard mean difference (SMD) and 95% confidence interval (CIs). Sensitivity analyses were conducted to exclude the mixed studies that might lead to potential bias. Publication bias was estimated using a funnel plot. All *P*-values were two-tailed, and significant statistical difference was considered when *P* values < 0.05.

## Results

### Study description

**Fig. 1:**
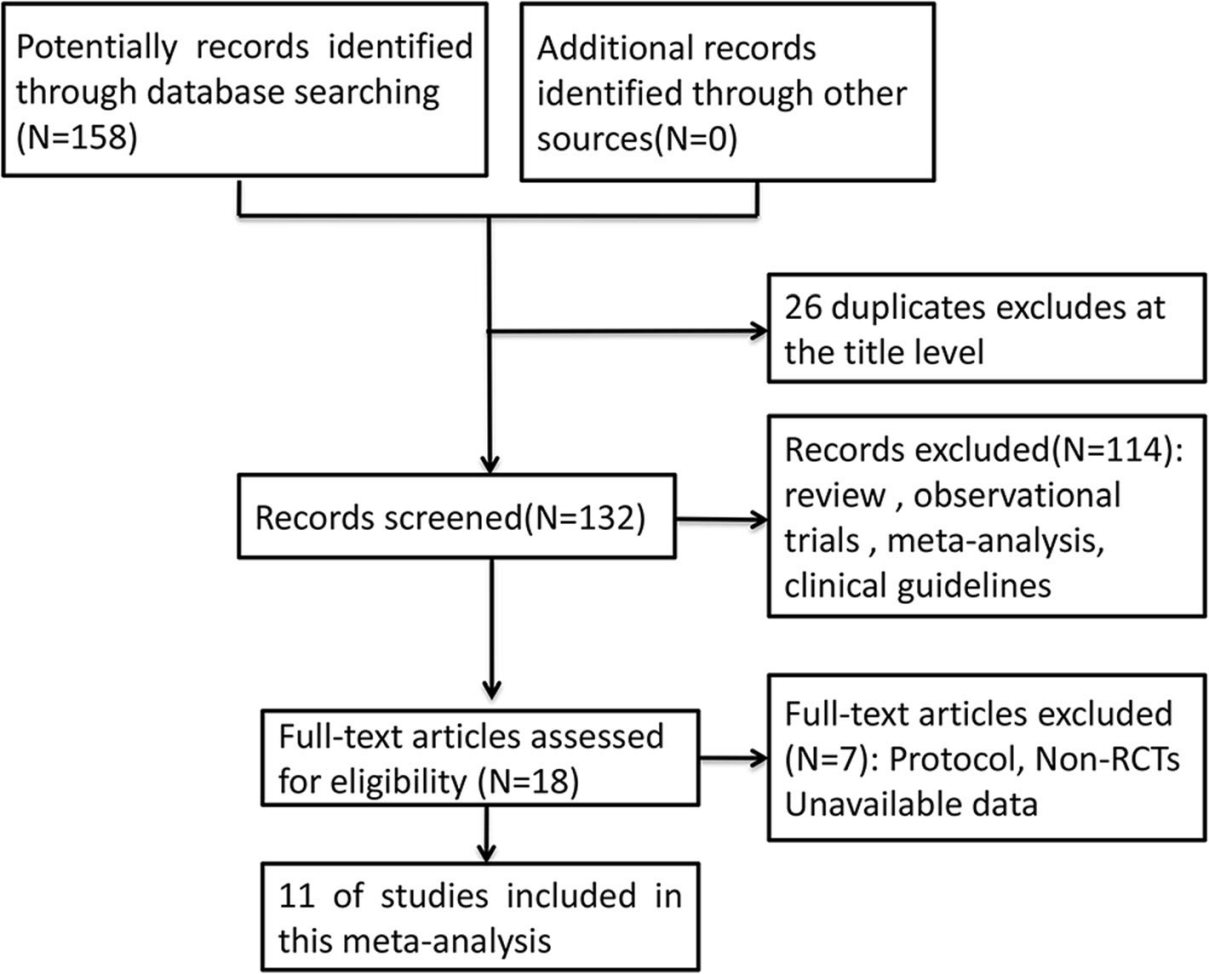
Flow diagram of studies identification and selection

The process of study screening was shown in Figs. 1, 158 potentially relevant articles were sifted out through electronic databases. After removing duplicates, review, observational trials, meta-analysis and clinical guidelines, 18 articles were full-text assessed for eligibility. Then 7 of these researches were excluded because of non-RCT and unavailable data. Finally, 11 trials [18–28] met the included criteria and were selected in this study. The characteristics of all included studies were shown in Table 1. A total of 1249 patients with DN were enrolled, 549 patients received lipoic acid plus valsartan combination therapy, 554 patients were treated with valsartan, and the remaining 146 patients were treated with lipoic acid. The ages of all participants ranged from 44.4 to 71.93 years old. The daily doses of lipoic acid were 600 mg, 450 mg or 300 mg, the dosage of valsartan was 80 mg, and the treatment duration was 14 days. Among these trials, 11 trials compared the effects of valsartan with that of lipoic acid plus valsartan on renal function, 4 trials compared the effects of lipoic acid plus valsartan with that of lipoic acid on UAER. Five studies were of high quality, while six studies were of low quality.

**Table 1:** Baseline characteristics of studies included in the meta-analysis

Author and year of publication	Study design	sample size			Age (mean ± SD)	Sex	Duration (days)	Dosage (mg)				Jadad scores
		LV	V	L	LV/V/L	male/female		L	V	Interventions	Outcomes	
Chen M, 201 8[18]	RCT	32	33	31	71.41 ± 7.52/ 70.92 ± 7.24/ 71.93 ± 7.05	48/48	14	600	80	LV/V/L	②④⑤⑥	3
Deng GJ,201 8[19]	RCT	40	40	40	47.3 ± 4.15/ 46.2 ± 5.89/ 47.8 ± 4.03	55/65	14	300	80	LV/V/L	①②	3
Huo Y, 201 4[20]	RCT	60	60	–	44.4 ± 4.2	57/63	14	600	80	LV/V	①④⑤⑥	4
Jiang ZY,201 9[21]	RCT	51	51	–	69.21 ± 3.54	59/43	14	600	80	LV/V	①③④	4
Li Y, 201 8[22]	RCT	43	43	–	63.21 ± 3.43/62.47 ± 3.65	52/34	14	600	80	LV/V	①②③	4
Lin XJ,201 3[23]	RCT	33	40	35	–	61/47	14	600	80	LV/V/L	①⑤⑥⑦	2
Ma L, 201 9[24]	RCT	48	48	–	57.26 ± 4.31/ 57.84 ± 3.92	56/40	14	600	80	LV/V	①③	4
Peng B, 201 3[25]	RCT	40	40	40	–	–	14	450	80	LV/V/L	①	3
Shi D, 201 8[26]	RCT	135	135	–	61.71 ± 2.52/ 61.56 ± 2.51	131/139	14	600	80	LV/V	①④⑤	3
Wen WB,201 9[27]	RCT	26	25	–	56 ± 11/56 ± 9	17/14	14	300	80	LV/V	①②	3
Zhao J, 201 7[28]	RCT	41	39	–	46.53 ± 13.35/45.78 ± 12.28	50/30	14	600	80	LV/V	①③④⑤⑥⑦	4

Note: RCT, randomized controlled trial; L, lipoic acid; V, Valsartan; LV, lipoic acid+ Valsartan; ①UAER = Urinary albumin excretion rate, ②Urinary albumin, ③β2-MG = β2-microglobulin, ④hs-CRP = Hypersensitive C-reactive protein, ⑤SOD = Superoxide dismutase, ⑥MDA = malondialdehyde, ⑦T-AOC = total antioxidant capacity

### Clinical outcomes

#### Urinary albumin excretion rate (UAER)

**Fig. 2:**
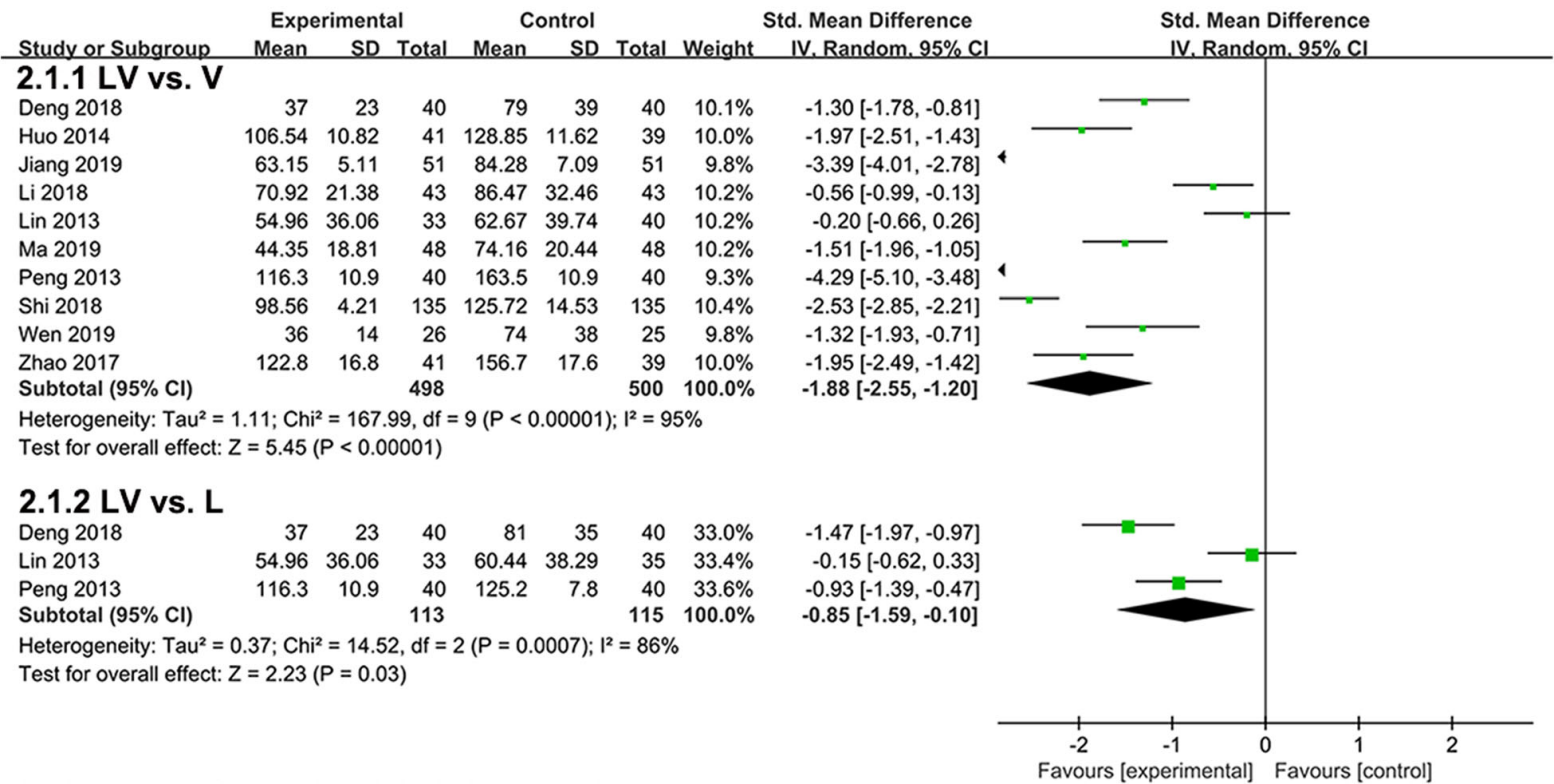
Comparison of the effects of lipoic acid plus valsartan with that of valsartan or lipoic acid monotherapy on UAER. LV, lipoic acid plus valsartan; L, lipoic acid; V, valsartan

Ten studies evaluated the effects of lipoic acid plus valsartan and that of valsartan monotherapy on UAER (Fig. 2). Significant heterogeneity existed between the two groups, and random effect model was used (*P* < 0.00001, *I*^*2*^ = 95%). Compared with valsartan monotherapy, treatment with lipoic acid plus valsartan could significantly reduce the UAER in patients with DN (*P* < 0.00001, SMD = -1.95, 95%CI = -2.55 to − 1.20). In addition, three studies compared the effects of lipoic acid plus valsartan with that of lipoic acid monotherapy on UAER. Random effect model was used because of marked heterogeneity (*P* = 0.0007, *I*^*2*^ = 86%). Treatment with lipoic acid plus valsartan was also superior to lipoic acid monotherapy in reducing UAER (*P* = 0.03, SMD = -0.85, 95%CI = -1.59 to − 0.1).

#### Urinary albumin

**Fig. 3:**
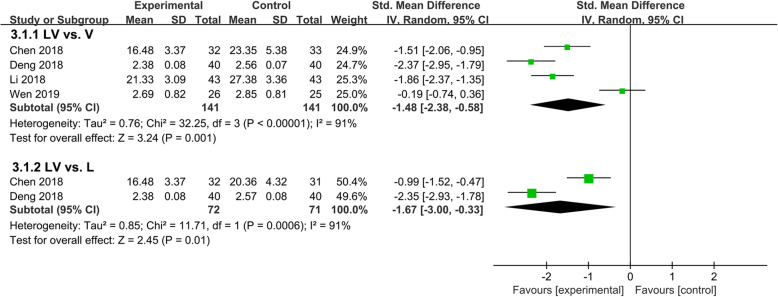
Comparison of the effects of lipoic acid combined with valsartan with that of monotherapy on the level of urinary albumin. LV, lipoic acid plus valsartan; L, lipoic acid; V, valsartan

Four trials involving 353 patients compared the effects of lipoic acid combined with valsartan with that of monotherapy on the level of urinary albumin. The results were shown in Fig. 3, significant heterogeneity between the two groups was observed, and random effect models were used (*P* < 0.00001, *I*^*2*^ = 91%; *P* = 0.0006, *I*^*2*^ = 91%). Compared with valsartan or lipoic acid monotherapy, lipoic acid combined with valsartan could remarkably decrease the level of urinary albumin (*P* = 0.001, SMD = -1.48, 95%CI = -2.38 to − 0.58; *P* = 0.01, SMD = -1.67, 95%CI = -3.00 to − 0.33).

#### β_2_-microglobulin (β_2_-MG) and hypersensitive C-reactive protein (hs-CRP)

**Fig. 4:**
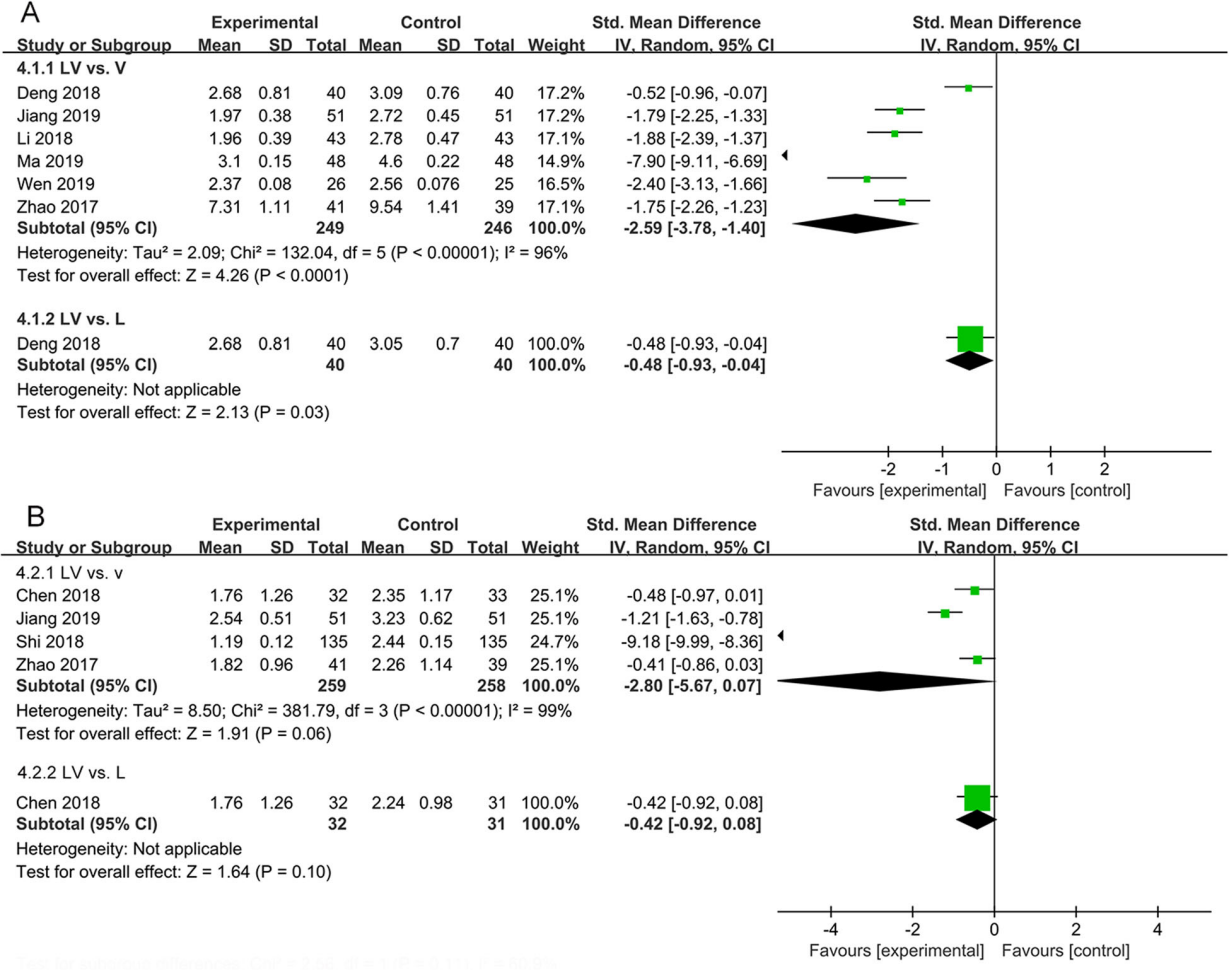
Comparison of the effects of lipoic acid combined with valsartan with that of monotherapy on β_2_-MG and hs-CRP. (A) the level of β_2_-MG; (B) the level of hs-CRP. LV, lipoic acid plus valsartan; L, lipoic acid; V, valsartan

Six studies evaluated the effects of lipoic acid combined with valsartan and that of monotherapy on the level of β_2_-MG in patients with DN. As shown in Fig. 4A, the pooled analysis indicated that heterogeneity was significant between the two groups (*P* < 0.00001, *I*^*2*^ = 96%), and a remarkable decline in the level of β_2_-MG in DN patients treated with lipoic acid plus valsartan was found (*P* < 0.001, SMD = -2.59, 95%CI = -3.78 to − 1.40; *P* = 0.03, SMD = -0.48, 95%CI = -0.93 to − 0.04).

Four studies involving 548 participants assessed the effects of lipoic acid combined with valsartan and that of monotherapy on the level of hs-CRP. We observed that there were no significant differences in hs-CRP level in DN patients receiving lipoic acid plus valsartan when compared with valsartan or lipoic acid monotherapy(*P* = 0.06, SMD = -2.80, 95%CI = -5.67 to 0.07; *P* = 0.10, SMD = -0.42, 95%CI = -0.92 to 0.08)(Fig. 4B).

#### The levels of oxidative stress

**Fig. 5:**
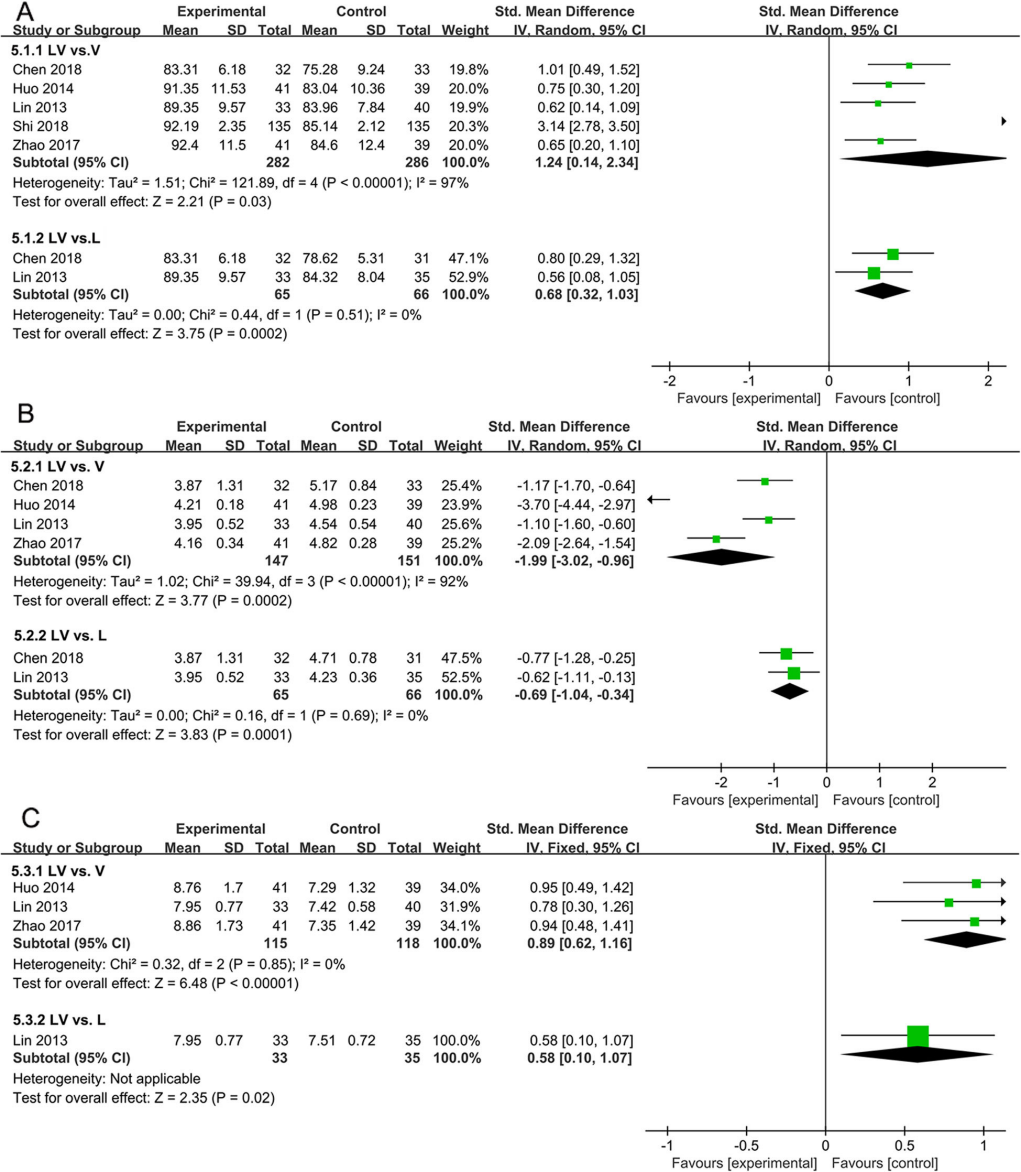
Comparison of the effects of lipoic acid plus valsartan with that of valsartan or lipoic acid monotherapy on oxidative stress. (A) the level of SOD; (B) the level of MDA; (C) the levels of T-AOC. LV, lipoic acid plus valsartan; L, lipoic acid; V, valsartan

Oxidative stress was one of the important mechanisms of diabetic nephropathy, and the levels of SOD, MDA and T-AOC in serum were all related to the process of renal impairments. Five trials evaluated the effects of lipoic acid combined with valsartan on the level of SOD in patients with DN (Fig. 5A). The results showed that lipoic acid combined with valsartan for the treatment of DN was much more effective in increasing the level of SOD than valsartan or lipoic acid monotherapy(*P* = 0.03, SMD = 1.24, 95%CI = 0.32 to 1.03; *P* = 0.0002, SMD = 0.68, 95%CI = 0.32 to 1.03). For MDA, the pooled results were showed in Fig. 5B. The effects of lipoic acid combined with valsartan treatments on the level of MDA were determined in four trials. Significant heterogeneity was observed among studies (*P* < 0.00001, *I*^*2*^ = 92%). Remarkable reduction of MDA was witnessed in patients received lipoic acid plus valsartan(*P* = 0.0002, SMD = -1.99, 95%CI = -3.02 to − 0.96; *P* = 0.0001, SMD = -0.69, 95%CI = -1.04 to − 0.34). Three studies with 268 patients assessed the effects of lipoic acid and valsartan combination on T-AOC. Statistical heterogeneity was not significant, and fixed effect model was applied (*P* = 0.85, *I*^*2*^ = 0%)(Fig. 5C). Compared with valsartan or lipoic acid monotherapy, lipoic acid plus valsartan could markedly increase T-AOC levels (*P* < 0.00001, SMD = 0.89, 95%CI = 0.62 to 1.16; *P* = 0.02, SMD = 0.58, 95%CI = 0.10 to1.07).

#### Sensitivity analysis and publication bias

**Fig. 6:**
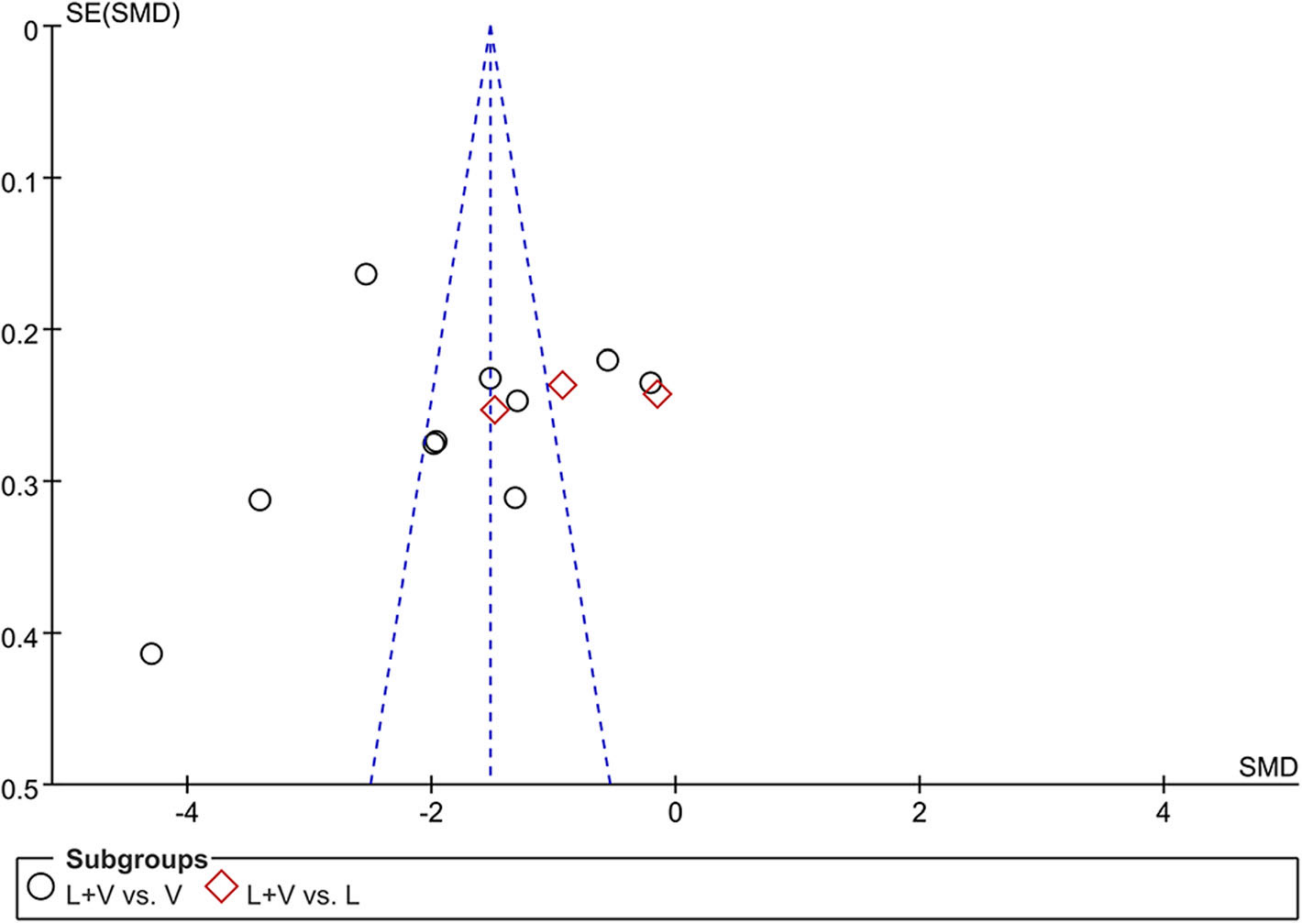
Funnel plot for evaluating the risk of bias in the meta-analysis. LV, lipoic acid plus valsartan; L, lipoic acid; V, valsartan

In the process of pooled analysis, we performed sensitivity analysis to evaluate heterogeneity. However, there was no significant difference in the value of *I*^*2*^, and the values of SMD and the overall effects were close when changing the analysis model from fixed effect model to random effect model. The results of publication bias were showed in Fig. 6, the funnel plot was not symmetrical, indicating the existence of potential publication bias.

## Discussion

DN is one of the major causes of end-stage renal failure, and the pathogenesis is related with the thickening of tubular and glomerular basement membrane, glomerulo-sclerosis and glomerular hypertrophy, endothelial dysfunction [29]. The main clinical feature of DN is the decline of glomerular filtration rate that leads to proteinuria [30]. At present, many clinical trials have been conducted to evaluate the effects of lipoic acid and valsartan used alone or in combination for the treatment of DN, but some of the results remained controversial. Therefore, we performed this meta-analysis to provide solid evidence for the selection of drugs for the treatment of DN. Moreover, as far as we know, this is the first systematic analysis to comprehensively evaluate the treating effects of lipoic acid combined with valsartan on renal functions.

Microalbuminuria is considered as an important marker for the diagnosis and assessment of DN progression [31]. In patients with DN, dysfunction of the glomeruli could boost urinary albumin excretion rate, cause excessive excretion of albumin in urine and bolster the production of microalbuminuria. However, renal function impairment also emerged among some diabetic patients in the absence of microalbuminuria [32]. Valsartan belongs to the class of angiotensin receptor antagonists, and is commonly used in the treatment of hypertension. It has certain protective effects on glomeruli by inhibiting angiotensin. Evidences from previous studies suggested that valsartan could remarkably lower the glomerular filtration rate and the production of proteinuria, playing a protective role in renal function in patients with type 2 diabetes [33, 34]. α-lipoic acid is an antioxidant that can protect β cells, lower the level of cholesterol, reduce proteinuria and mesangial expansion in diabetic mice, thus inhibiting the development and progression of DN [35]. Additionally, α-lipoic acid combined with alprostadil could decrease the levels of serum inflammatory factors and improve renal function of patients with DN [36]. In our study, the pooled analysis indicated that lipoic acid combined with valsartan was superior in lowering UAER, the level of urinary albumin and β_2_-MG when compared with lipoic acid or valsartan monotherapy for the treatment of DN. The results were consistent with previous study [21].

Oxidative stress is a kind of stress response when the body is subjected to various harmful stimuli, and eventually causes damage to various cells, tissues and organs of the body. Current research found that oxidative stress was associated with the pathophysiology of DN [37]. Persistent hyperglycaemia caused the overproduction of reactive oxygen species (ROS) and activation of inflammatory mediators, inhibiting antioxidant defense mechanisms and eventually leading to oxidative stress which resulted in injury in the vessels and kidneys of diabetic patients [38]. Moreover, oxidative stress could lead to the abnormalities of hematological indices which might be used to predict the progression of DN. MDA and advanced oxidation products of protein (AOPP) as the peroxidation products of lipids and proteins could reflect the level of oxidative stress, and SOD and T-AOC are indicators that reflect the antioxidant capacity of the body [39]. Other study showed that valsartan could reduce the level of MDA and 8-hydroxy-deoxy guanosine, inhibit cyclosporine-A induced oxidative stress and alleviate the renal damages [40].

Lipoic acid acts as an antioxidant that can reduce oxidative stress, and it also has significant therapeutic effects on cardiovascular diseases, diabetes and its complications and neurodegenerative diseases. In alloxan-induced diabetic rabbits, low doses of lipoic acid significantly lowered the concentration of urine albumin, ameliorated oxidative stress and renal injury, so the agent proved to be effective in the treatment of diabetes and DN [16]. Later, another study further confirmed that the levels of urinary MDA and creatinine (Cr) rose significantly, the ratios of SOD and serum glutathione peroxidase (SGSH-Px) were markedly decreased in patients with diabetes; α-lipoic acid could significantly lower the ratios of MDA and Cr, elevate the levels of SOD and SGSH-Px by inhibiting oxidative stress, and provide protection against glomerular podocyte injury [41]. In the present study, our results indicated that lipoic acid combined with valsartan could significantly increase the levels of SOD and T-AOC, decrease the level of MDA when compared with valsartan or lipoic acid monotherapy. From these results, we inferred that combination therapy of lipoic acid and valsartan inhibited oxidative stress, and enhanced antioxidant capacity.

Although the combination therapy proved to be more effective than each of the monotherapy, several possible limitations should still be taken into consideration to facilitate deeper research in the future. First, most of these included trials had relatively low sample sizes, which might be insufficient and our conclusions might not be flawless. Second, significant heterogeneity was observed between these studies, and sensitivity analysis was used to determine potential heterogeneity. Third, potential publication bias was existed, which might affect the accuracy of the results. Therefore, RCTs with large sample size and high quality were still needed to further confirm these conclusions.

## Conclusions

Overall, eleven trials were included in this meta-analysis to assess the effects of lipoic acid combined with valsartan on renal functions in patients with DN. The pooled findings indicated that lipoic acid combined with valsartan could significantly reduce the level of urinary albumin and oxidative stress, enhance antioxidant capacity and mitigate renal function damages in patients with DN, and the findings proved to be helpful references for the selection of treatment drugs for DN.

## Data Availability

All data generated or analyzed during this study are included in this published article, and you can find these data in references [18–28].

## Abbreviations

DN: Diabetic nephropathy
RCTs: Randomized controlled trials
DM: Diabetes mellitus
SGLT2: Sodium-glucose cotransporter 2
UAER: Urinary albumin excretion rate
β_2_-MG: β_2_-microglobulin
hs-CRP: Hypersensitive C-reactive protein
MDA: Malondialdehyde
T-AOC: Total antioxidant capacity
ROS: Reactive oxygen species
